# Regulation of Gram-Positive Conjugation

**DOI:** 10.3389/fmicb.2019.01134

**Published:** 2019-05-22

**Authors:** Verena Kohler, Walter Keller, Elisabeth Grohmann

**Affiliations:** ^1^ Institute of Molecular Biosciences, BioTechMed Graz, University of Graz, Graz, Austria; ^2^ Life Sciences and Technology, Beuth University of Applied Sciences Berlin, Berlin, Germany

**Keywords:** Gram-positive bacteria, type IV secretion system, conjugation system, regulation, plasmid, integrative and conjugative element

## Abstract

Type IV Secretion Systems (T4SSs) are membrane-spanning multiprotein complexes dedicated to protein secretion or conjugative DNA transport (conjugation systems) in bacteria. The prototype and best-characterized T4SS is that of the Gram-negative soil bacterium *Agrobacterium tumefaciens*. For Gram-positive bacteria, only conjugative T4SSs have been characterized in some biochemical, structural, and mechanistic details. These conjugation systems are predominantly encoded by self-transmissible plasmids but are also increasingly detected on integrative and conjugative elements (ICEs) and transposons. Here, we report regulatory details of conjugation systems from *Enterococcus* model plasmids pIP501 and pCF10, *Bacillus* plasmid pLS1, *Clostridium* plasmid pCW3, and staphylococcal plasmid pSK41. In addition, regulation of conjugative processes of ICEs (ICE*Bs*1, ICE*St*1, ICE*St*3) by master regulators belonging to diverse repressor families will be discussed. A special focus of this review lies on the comparison of regulatory mechanisms executed by proteins belonging to the RRNPP family. These regulators share a common fold and govern several essential bacterial processes, including conjugative transfer.

## Introduction

Horizontal gene transfer (HGT) is leadingly involved not only in the evolution of bacteria but also in the dissemination of antibiotic resistances and pathogenicity determinants. This process can be subdivided into three mechanisms: transformation, transduction, and conjugation (Daubin and Szöllősi, 2016), with the latter being the most common type involved in spreading of traits that are beneficial under distinct environmental conditions (Davies and Davies, 2010; Sultan et al., 2018). Conjugative transport of DNA from a donor to a recipient cell requires direct cell-to-cell contact and the formation of a pore, where the DNA molecule can be transported through (Perry and Wright, 2013). Conjugation has been described over large taxonomic distances between unrelated bacterial species (Tamminen et al., 2012). Two different conjugative mechanisms are known to date: the transport of single-stranded (ss) DNA, found in both Gram-positive (G+) and Gram-negative (G−) systems vs. the transport of double-stranded DNA. The second mechanism has been described for G+ actinomycetes, and recent work summarizes this process extensively (Thoma and Muth, 2016; Pettis, 2018). Factors needed for conjugative processes can be encoded on plasmids or integrative and conjugative elements (ICEs). The basic mechanism of conjugation is conserved in most G+ conjugative systems, which facilitate the transport of ss-DNA *via* a molecular machinery encoded by multiple genes that are mostly organized in a single operon. These systems comprise a relaxase, a coupling protein and a mating pair formation (MPF) complex. Relaxases are essential factors in the process of conjugative transfer. They initiate the process by site- and strand-specific cleavage at the *nic*-site of the origin of transfer (*oriT*), forming a covalent complex with the cleaved DNA. For G+ systems, only two relaxases have been structurally characterized so far (Edwards et al., 2013; Pluta et al., 2017). Together with potential accessory factors, the relaxase-DNA complex is called the relaxosome. The coupling protein brings the relaxosome to the MPF complex that forms the actual channel. Conjugation systems usually consist of several mating pair formation proteins, one or more ATPases and proteins facilitating the contact with recipients. In contrast to G-systems that rely on conjugative pili, the contact between donor and recipient is formed *via* surface adhesins in G+ systems. Simultaneously with transfer processes, DNA replication ensures that both donor and new host have a double-stranded version of the plasmid or ICE (Guglielmini et al., 2011; Grohmann et al., 2017).

Transfer of DNA *via* conjugative processes needs to be stringently regulated to reduce the metabolic burden on the host (Koraimann and Wagner, 2014; Singh and Meijer, 2014). Thus, gene products required for conjugation are either kept in a default “OFF” state and are induced by signaling molecules from potential recipients/the environment or conjugative genes are constitutively produced at low abundance to keep fitness costs for the host at a minimum (Frost and Koraimann, 2010; Bañuelos-Vazquez et al., 2017; Stingl and Koraimann, 2017). In this review, we will summarize the current knowledge on the regulation of conjugative processes, focusing on selected conjugation systems from G+ bacteria.

## Plasmids vs. Integrative and Conjugative Elements: Similarities and Differences

**Figure 1:**
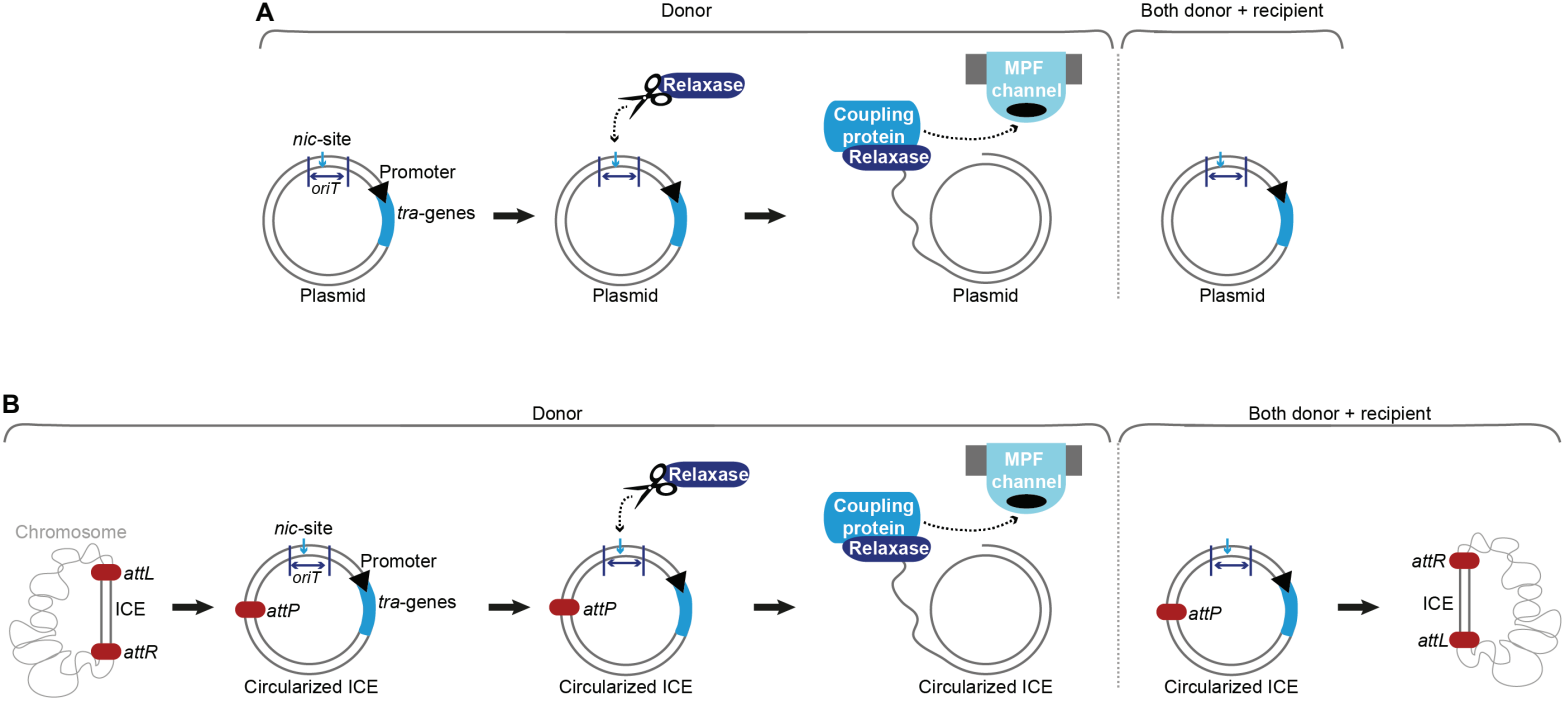
Conjugation of plasmids and integrative and conjugative elements (ICEs). **(A)** Upon a signal (internal or external), the relaxase introduces a single-strand (ss) break at the *nic*-site of the origin of transfer (*oriT*) in the donor cell and covalently binds the ss-DNA. The coupling protein interacts with the relaxosome (relaxase, accessory factors and ss-plasmid DNA) and brings the relaxase-DNA complex to the mating pair formation (MPF) channel. The ss-DNA (most likely covalently bound by the relaxase) is shuttled to the recipient cell. In parallel, replication processes ensure that both donor and recipient harbor a double-stranded version of the conjugative plasmid. **(B)** With the aid of an excisionase and an integrase, the integrative and conjugative element (ICE) is excised from the host’s chromosome at *attL* and *attR* attachment sites, forming an *attP* site on the circularized ICE and an *attB* site on the bacterial chromosome. Processing of the DNA, transport and replication follow a comparable mechanism as described for conjugative plasmids in **(A)**. After successful conjugation and replication, the ICE is again integrated into the host’s chromosome.

Conjugative plasmids and ICEs harbor all necessary genetic information for conjugative transfer processes (Bañuelos-Vazquez et al., 2017). The principal difference between conjugative plasmids and ICEs lies in their respective maintenance mechanisms within a bacterial cell. While plasmids replicate autonomously, ICEs must integrate into bacterial chromosomes for stable inheritance (Figure 1; Perry and Wright, 2013; Burrus, 2017).

Plasmids are autonomously replicating elements that can be categorized into incompatibility (Inc) groups according to their replication and partitioning systems. The spreading of plasmids between unrelated genera is involved in the emergence of antibiotic-resistant bacteria (Sultan et al., 2018). These elements generally carry non-essential genetic features, which might become important under distinct environmental conditions, e.g. in the presence of antibiotic selection pressure (Bañuelos-Vazquez et al., 2017). Plasmids that carry all necessary factors for mobilization and transfer processes are denoted as self-transmissible or conjugative. Biofilm formation plays a substantial role in transfer and dissemination of conjugative plasmids. Conjugative transfer was shown to be considerably higher in biofilms (Kelly et al., 2009).

ICEs are omnipresent in bacterial genomes and were found to be the most abundant conjugative elements in prokaryotes (Ghinet et al., 2011; Guglielmini et al., 2011; Guédon et al., 2017). The exact processes of ICE conjugation are not completely elucidated. It is supposed that these events resemble the ss-plasmid DNA shuttling *via* conjugation systems encoded on plasmids. Since two additional steps, excision and re-integration, are required, ICEs harbor genes that resemble factors of lysogenic phages (Wozniak and Waldor, 2010). These elements show a modular structure with genes of the same/similar function clustered together and usually consist of a maintenance module (responsible for integration and excision), a dissemination module (required for conjugative transfer), and a regulation module (Burrus and Waldor, 2004). An integrated ICE shows a behavior reminiscent of prophages, with most mobility genes suppressed and passively inherited along with the chromosome. Depending on the ICE family, an intra-/intercellular/environmental signal triggers its excision and formation of a circular plasmid-like form, serving as a substrate for the conjugative transfer machinery. After successful transport, the ICE re-integrates into the recipient’s chromosome. Integration into and excision from the host chromosome are catalyzed by dedicated enzymes. An integrase (frequently a tyrosine recombinase) governs the reaction between a sequence of the recombination module of the ICE (*attP*) and a sequence on the host chromosome (*attB*), yielding the attachment sites (*attL* and *attR*) that border the ICE after successful integration. In most cases, an excisionase aids in reverting this reaction, forming again an *attP* site on the circularized ICE and an *attB* site on the host’s chromosome. As plasmids, ICEs also harbor genes beneficial for their host under specific conditions, e.g. mediating resistance to antimicrobial drugs, heavy metals, and infections by phages (Burrus and Waldor, 2004; Burrus, 2017).

## Selected Mobile Genetic Elements and Their Regulation of Conjugative Processes

The following sections concentrate on selected plasmids or ICEs from different G+ species, ranging from broad-host range plasmids that produce their conjugative systems constitutively at low levels to inducible/repressible plasmids responding to stimuli from small peptides, called pheromones or autoinducers. These small peptides frequently regulate cellular signaling processes according to the population density, a process denoted as quorum sensing (QS). QS is described to govern essential processes, like virulence, sporulation, and gene transfer. It enables bacteria to sense information about the surrounding species composition and to adapt their expression profiles. This process encompasses the production, release, and detection of autoinducers, the latter is done by a specific sensor component (Papenfort and Bassler, 2016). G+ bacteria usually use oligopeptides as signaling molecules, and the receptor protein interacts directly with the signaling peptide (Monnet et al., 2016). Once a certain level of the pheromone (the quorum) in the external environment is reached, a target sensor kinase or a response regulator is either activated or repressed. Downstream processes then lead to altered expression of QS-dependent genes (Rocha-Estrada et al., 2010).

Pheromone-sensor receptors of the RRNPP-family are found in distantly related G+ bacteria among others on plasmids and ICEs harboring conjugation systems (e.g. pCF10, pLS20 and ICE*Bs*1) (Perez-Pascual et al., 2016). This protein family is named after its prototypical members, Rap, Rgg, NprR, PlcR, and PrgX that despite low sequence homology displays remarkable structural similarities. The defining feature of this group is the C-terminal domain that directly interacts with the pheromone and forms a tetratricopeptide repeat (TPR) domain-like conformation. Structural characterization of these receptors revealed a right-handed super-helical architecture, where the respective ligand is bound to an inner concave binding site of the helix-turn-helix (HTH) repeats (Zeytuni and Zarivach, 2012; Do and Kumaraswami, 2016).

An additional unifying feature of these sensor receptors is the structure of the secreted signaling peptides that are usually linear, 5–10 amino acids long, unmodified, and produced from a longer precursor protein. Pheromones are synthesized *via* the conventional path of ribosomal translation, processing/cleavage, and secretion. Since RRNPP sensor receptors are present inside a cell, the secreted pheromone must be taken up *via* oligopeptide permeases. These enzymes are sometimes aided by accessory proteins that provide high selectivity for the respective peptide (e.g. PrgZ of pCF10; Leonard et al., 1996; Neiditch et al., 2017).

### Sex-Pheromone Responsive Plasmid pCF10 From *Enterococcus faecalis*

An increasing number of clinical *E. faecalis* isolates carry conjugative plasmids that are transferred upon induction of peptide pheromones and code for antibiotic resistances (Dunny and Berntsson, 2016). Sex-pheromone responsive plasmids include the tetracycline-resistance plasmid pCF10, one of the best-characterized representatives of this plasmid family, and pAD1. Both plasmids serve as model systems for pheromone-responsive conjugative systems in enterococci (Chen et al., 2017).

*prgQ* is the conjugative operon of pCF10. It consists of three cassettes. One cassette encodes three surface adhesins required for contacting recipients and *prgU* (Bhatty et al., 2017), encoding a regulator that will be described in further details below. The second cassette harbors the Prg/Pcf MPF and the third cassette codes for factors required for processing the pCF10 plasmid DNA. Several transcriptional and post-transcriptional processes regulate the expression of the *prgQ* operon to guarantee strict control of the Prg/Pcf conjugation system assembly and conjugative transfer (Johnson et al., 2010).

**Figure 2:**
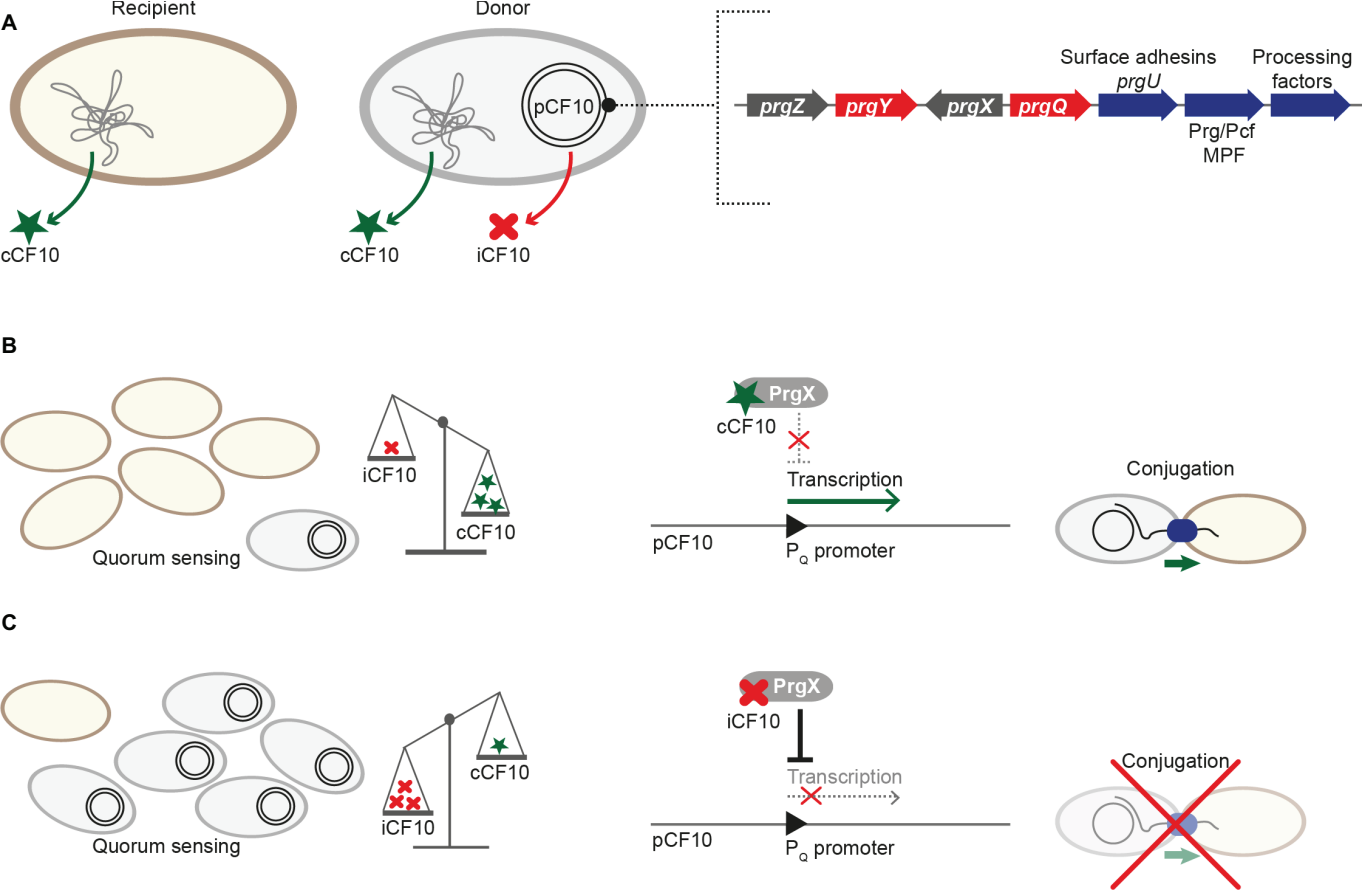
Regulation of pCF10 conjugative transfer. **(A)** While both recipient and donor cell produce the pheromone cCF10 from *ccfA* on the chromosome, only cells harboring pCF10 secrete the inhibitor iCF10 that is produced from *prgQ*. *prgQ* lies upstream of the three cassettes responsible for conjugative transfer: The first cassette codes for the surface adhesins PrgA, PrgB, and PrgC and the regulator PrgU, the second harbors members of the Prg/Pcf conjugation system, and the third is composed of genes for processing factors (including the relaxase). The RRNPP-family protein PrgX is the master regulator and PrgY aids in reducing cCF10 pheromone activity. *prgZ* codes for a permease that imports both cCF10 and iCF10. **(B)** The relative ratio between cCF10 and iCF10 increases with a higher proportion of potential recipients present. This leads to increased import of cCF10, which binds to PrgX, thus interfering with its ability to repress the P_Q_ promoter. Transcription of genes required for conjugative transfer is activated, and conjugation takes place. **(C)** The cCF10/iCF10 ratio decreases with a lower proportion of potential recipients present, which leads to increased import of the inhibitor peptide iCF10. iCF10-PrgX complexes repress the P_Q_ promoter, thus downregulating transcription of genes required for conjugative transfer and ultimately inhibiting conjugation.

**Figure 3:**
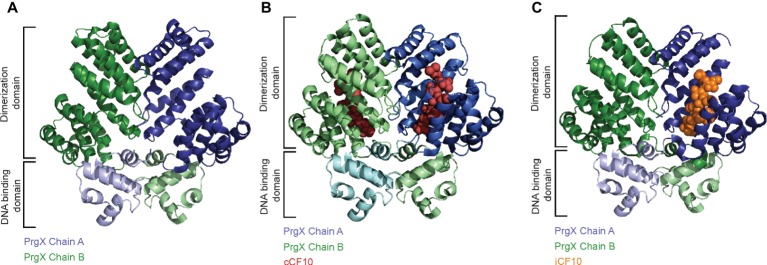
Structural basis for the regulation of the P_Q_ promoter. **(A)** The PrgX protein forms tetramers, which repress transfer gene expression by binding to two adjacent operator sites, one of which overlaps with the P_Q_ promoter. The tetramer is built as a dimer of dimers with each dimer occupying one operator site with the N-terminal DNA-binding domain, whereas the C-terminal domain accommodates the dimerization and pheromone-binding function. The dimer of apo-PrgX (PDB: 2AXU) is shown in a cartoon presentation with the chains colored in blue and green, respectively. **(B)** Binding of the pheromone cCF10 (red spheres) leads to a conformational change in the C-terminal region of PrgX (PDB: 2AXZ), alleviating the repression of the P_Q_ promoter. **(C)** A surplus of iCF10 (orange spheres), which competes with cCF10 for the same binding site (PDB: 2GRL), reinstates the repressed state of the P_Q_ promoter.

The transcriptional regulator PrgX that belongs to the RRNPP-family binds to the P_Q_ promoter and represses transcription (Figure 2A; Nakayama et al., 1994; Kozlowicz et al., 2004). PrgX further controls its own expression. Unlike other QS-systems (including the Rap-Phr cassettes from pLS20 and ICE*Bs1*), the signal that is sensed by PrgX originates from two different cell types (donor and recipient). This enables the plasmid donor to control conjugation in response to recipient population density (Kozlowicz et al., 2006). PrgX can bind two different heptapeptides, the inducer peptide cCF10 produced from *ccfA* on the bacterial chromosome and the inhibitor iCF10 that is encoded on pCF10 plasmid solely in donor cells (Antiporta and Dunny, 2002). Both inducer and inhibitor peptides are produced by cleavage of precursor proteins, secreted, and imported by the peptide-binding protein PrgZ and chromosomally encoded permeases (Leonard et al., 1996). Inside cells, these pheromones compete for PrgX binding, as both bind to the same cleft within the PrgX C-terminal dimerization domain while interacting with different residues (Figure 3; Shi et al., 2005). Interestingly, in contrast to canonical transcription factors that are usually low abundant and modulated by higher-abundant ligands, pCF10-harboring cells usually display an excess of PrgX (15-fold excess of PrgX to its binding site), while the pheromones are present at low concentrations (Mori et al., 1988; Nakayama et al., 1994; Caserta et al., 2012). While its apo-form is indeed able to bind DNA and repress P_Q_ transcriptional activity at high protein concentrations, PrgX complexed with both the inducer peptide as well as the inhibitor peptide leads to shifted/supershifted DNA complexes with much higher affinities than the unbound form (Caserta et al., 2012; Chen et al., 2017). Thus, following the import of inhibitor/inducer peptide, DNA-bound apo-PrgX is replaced by its complexed form. It is further suggested that the binding affinity of the pheromone (both inhibitor and inducer peptide) for PrgX is considerably stronger than that of complexed PrgX to its DNA binding site. Thus, changes of the donor’s induction state most likely come from the exchange of the PrgX apoform for a complexed PrgX form on the DNA (Chen et al., 2017). X-ray crystallography revealed that PrgX exists as a tetramer formed by two dimers. PrgX dimers bind to two operators present on pCF10, O1 and O2. Thus, it has been proposed that the two dimers bound to O1 and O2 interact with each other and form a stable DNA loop. This DNA loop restrains the RNA polymerase from accessing the *prgQ* promoter. When cCF10 binds to the C-terminal domain of PrgX, conformational changes of PrgX are induced. These structural alterations are suggested to break up the tetramers, thus allowing the polymerase to bind to the *prgQ* promoter. By contrast, while iCF10 is thought to compete with cCF10 for the binding site, the inhibitor peptide most likely does not induce structural changes and the tetramer should not be destabilized (Shi et al., 2005). Since the iCF10 precursor is encoded within the *prgQ* locus, enhanced transcription from the *prgQ* promoter increases iCF10 levels, resulting in repression of the transcription of the *prgQ* locus (Kozlowicz et al., 2006). Interestingly, in contrast to other Rap protein-dependent pheromones, neither cCF10 nor iCF10 harbors a positively charged amino acid at the second position (Rocha-Estrada et al., 2010).

In the absence of inducer pheromone sensing, several mechanisms control transcription of the *prgQ* operon (Bae et al., 2004). These comprise PrgX-mediated repression of the P_Q_ promoter, elevated PrgX repression by binding of the small inhibitor peptide iCF10 that is expressed from a gene directly downstream of the P_Q_ promoter and production of *anti-Q*, an antisense RNA produced from the convergent P_X_ promoter, that binds *prgQ* transcripts and induces formation of a termination structure, which blocks transcriptional elongation of *prgQ* transcripts (Nakayama et al., 1994; Shokeen et al., 2010; Chatterjee et al., 2013). Uninduced donor cells show transcriptional activity of the *prgQ* promoter, leading to short (approximately 380 nucleotide long) transcripts. Upon cCF10 binding to PrgX and destabilization of the PrgX tetramer, the number of short *prgQ* transcripts increases, leading to transcription of the whole operon. Within the first 30 min of cCF10 pheromone-exposure, donor cells synthesize the Prg/Pcf conjugation system, form intercellular aggregates due to production of PrgB, one of the surface adhesins, and transfer pCF10 at high frequencies with up to one transconjugant per recipient (Figure 2B). In the following 1–2 h, *prgQ* transcription returns to pre-induction levels (Hirt et al., 2005; Chatterjee et al., 2013). Two processes ensure that pCF10-harboring cells do not undergo self-induction. PrgY, a plasmid-encoded membrane protein, reduces pheromone activity produced by donor cells. The extracellular domain of PrgY was proposed to interact with and modify/degrade cCF10 heptapeptides, thus reducing endogenous pheromone activity in donor cells (Chandler et al., 2005). Residual pheromone activity is neutralized by the inhibitor peptide iCF10 encoded by *prgQ* (Nakayama et al., 1994). This inhibitor not only plays a crucial role in returning induced donor cells to the pre-induction state but also serves as a sensor of donor cell density (Figure 2C; Chatterjee et al., 2013). After 30–60 min, iCF10 levels reach a certain threshold and consequently reduce transcriptional activity to pre-induction levels. Genes located between *prgQ* and *prgA* (e.g., *prgR*, *prgS*) code for factors modulating transcription and translation of genes required for conjugation (Chung and Dunny, 1992; Bensing et al., 1997).

It was recently demonstrated that cCF10 induction is highly toxic for cells without *prgU*, a small gene downstream of *prgB*, encoding an essential surface adhesin (Bhatty et al., 2017). These *prgU* mutants displayed impaired cell envelope integrity and overproduction of Prg adhesins. By contrast, PrgU overproduction rendered cells insensitive to the sex pheromone and blocked surface adhesin production. PrgU was found to belong to a novel class of RNA binding regulators, reducing toxicity by overproduced surface adhesins. Thus, PrgU exerts another layer of negative regulation of pCF10 conjugative processes. Modeling studies showed that PrgU most likely exists as a tetramer and comprises a PUA (pseudouridine synthase and archaeosine transglycosylase) fold, domains widely distributed and usually interacting with RNA substrates (Pérez-Arellano et al., 2007). Thus, it is hypothesized that PrgU controls Prg adhesin production by binding of RNA substrates, likely regulating trans-acting sRNAs or *prgQ* transcripts. Interestingly, *prgB-prgU* gene pairs were identified in many *E. faecalis* strains and several other enterococci and staphylococci, suggesting that this genetic linkage has evolved to regulate the production of PrgB-like adhesins (Bhatty et al., 2017).

### pLS20 From *Bacillus subtilis*

**Figure 4:**
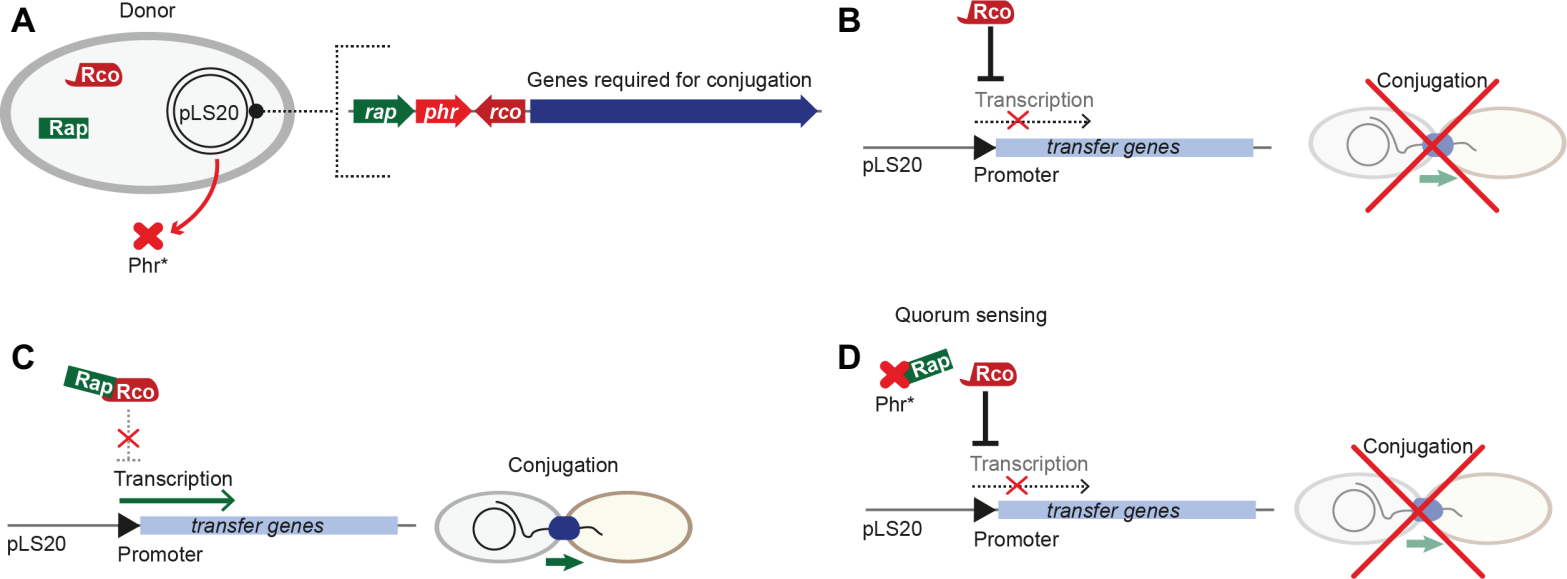
Regulation of pLS20 conjugative transfer. **(A)** Key players involved in regulation are encoded on the pLS20 plasmid, including the master regulator Rco_LS20_ and the Rap_LS20_-Phr_LS20_ sensor-pheromone cassette upstream of genes required for conjugation. **(B)** The master regulator Rco_LS20_ represses transcription of transfer genes, thus ultimately inhibiting conjugation. **(C)** Binding of Rap_LS20_ to the master regulator Rco_LS20_ leads to conformational changes of Rco_LS20_, interfering with transcriptional repression of transfer genes, thus conjugation can take place. **(D)** Upon a distinct pheromone concentration (the quorum), the pheromone Phr*_LS20_ interacts with Rap_LS20_ and interferes with its binding to the master regulator Rco_LS20_. In consequence, Rco_LS20_ represses transcription of transfer genes and inhibits conjugation.

pLS20 is a 65-kbp conjugative plasmid originally isolated from *B. subtilis natto.* It was shown to considerably influence the physiology of its host, e.g. by inhibition of natural competence by the plasmid-encoded repressor ComK (Singh et al., 2012). An operon encoding more than 40 genes responsible for conjugative processes lies downstream of a divergently oriented gene, encoding Rco_LS20_, the master regulator that keeps conjugative processes in a default “OFF” state, reminiscent of PrgX from pCF10 (Figure 4A). Activation of conjugation requires the RRNPP-family protein Rap_LS20_, the anti-repressor, which is regulated by the signaling pentapeptide Phr*_LS20_. Conjugative transfer of pLS20 takes place only during exponential growth (Itaya et al., 2006). pLS20-encoded conjugative proteins are regulated on three levels: first, expression of conjugative proteins and the key transcriptional regulator Rco_LS20_ is controlled by two overlapping divergent promoters of different strengths. Second, Rco_LS20_ exerts three different functions. It is not only a repressor of the main promoter but also an autoregulator of its own promoter, either negatively or positively depending on its abundance. Third, a DNA loop is formed by binding of tetrameric Rco_LS20_ to two operators, overlapping with the divergent promoters (Figure 4B; Ramachandran et al., 2014). This scenario is similar to that described for PrgX in the pCF10 system. In contrast to sex-pheromone responsive plasmids like pCF10, pLS20 is not activated by recipient’s signaling (*via* an activator peptide like cCF10), but by factors encoded on the plasmid itself. A Rap-Phr module regulates the transfer of pLS20 (Singh et al., 2013). Most Rap-Phr cassettes known to date govern processes like sporulation, competence, or protease/antibiotic synthesis, where the RRNPP-family protein Rap inhibits these events by interacting with a factor required for activating genes involved in these processes. The *phr* gene codes for a protein that is processed into a penta/hexapeptide after Sec-dependent secretion. Following re-uptake, the peptide binds and inactivates its cognate Rap protein (Pottathil and Lazazzera, 2003). The anti-repressor Rap_LS20_ directly binds to the helix-turn-helix domain of Rco_LS20_ in an equimolar stoichiometry, thus most likely interfering with DNA binding of Rco_LS20_ (Figure 4C). The pheromone Phr*_LS20_, produced as a precursor molecule, directly interacts with Rap_LS20,_ inducing a conformational change of this regulator, which leads to dissociation from Rco_LS20_ (Figure 4D). This pheromone is secreted and re-imported into the cells; thus, it is a signal that underlies cell density (Rösch and Graumann, 2015). Interestingly, in contrast to pheromone-induction of pCF10 and processes where QS activates gene expression, genes required for pLS20 conjugation are repressed when the signaling molecule produced by the conjugative plasmid itself reaches a certain quorum. Consequently, when a large number of donor cells is present in the population and thus high levels of the pheromone Phr*_LS20_, conjugative processes are inhibited, while plasmid dissemination is activated when more recipient cells are around (and thus lower pheromone levels; Singh and Meijer, 2014). Further, Phr*_LS20_ plays an essential role in returning conjugative processes to the default “OFF”-state (Singh et al., 2013). This results in heterogeneity of the bacterial population, where up to 30% of the cells induce expression of the operon responsible for conjugation. Several other plasmids, including pX01 of *B. anthracis*, and pBS32 as well as pTA1060 from *B. subtilis*, carry Rap-Phr modules, pointing toward a similar mode of regulation (Koetje et al., 2003; Bongiorni et al., 2006; Parashar et al., 2013).

### ICE*Bs*1 From *Bacillus subtilis*

**Figure 5:**
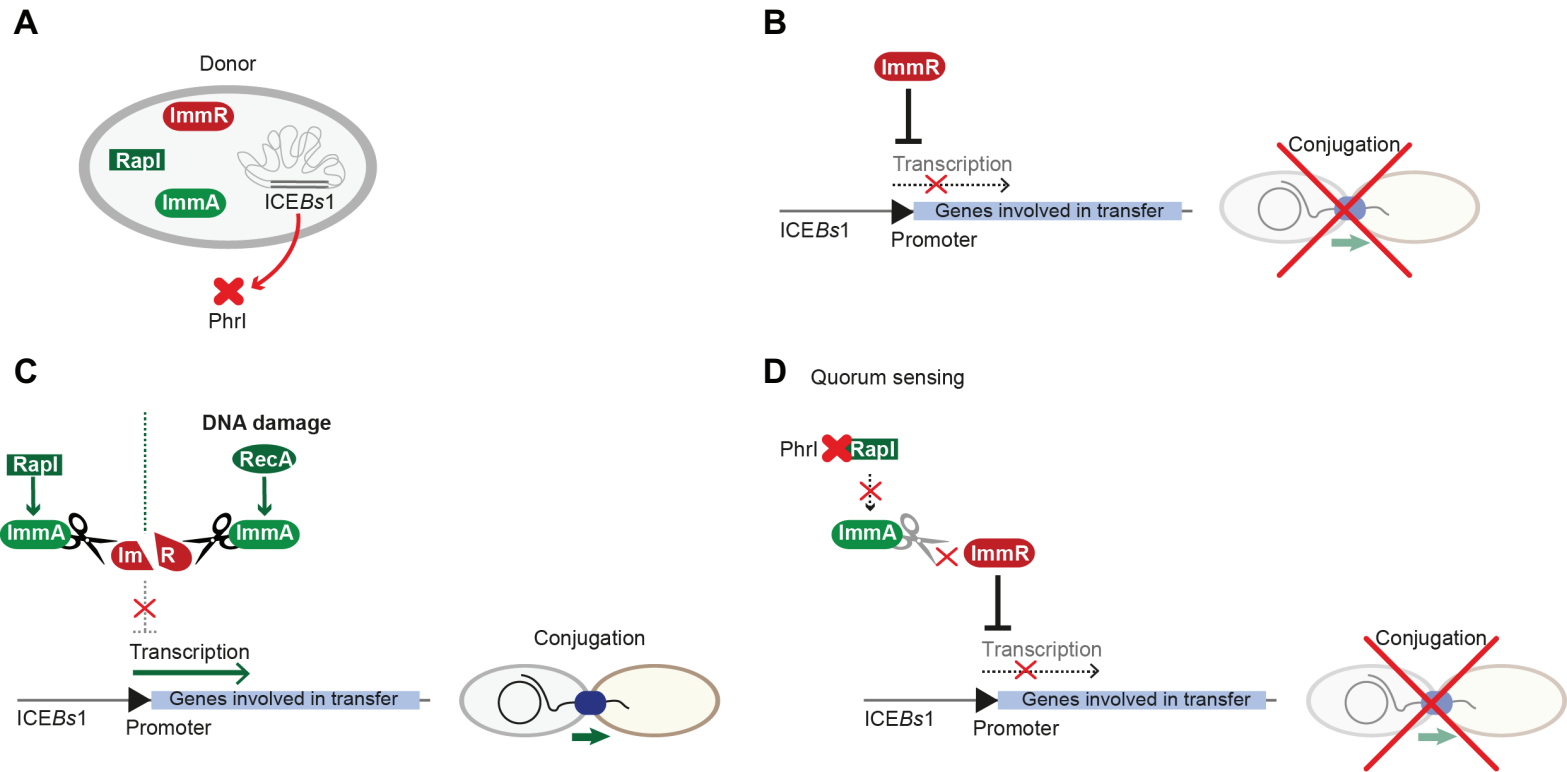
Regulation of ICE*Bs*1 conjugative transfer. **(A)** ICE*Bs*1 harbors most key players involved in regulation of conjugative processes, including the master-regulator ImmR, the metalloprotease ImmA, and the RapI-PhrI sensor-pheromone cassette. **(B)** Similar to processes in pLS20, the master-regulator ImmR represses transcription of genes involved in mobilization and transfer, which ultimately leads to inhibition of conjugation. **(C)** RapI or RecA that is activated by general DNA damage control proteolytic cleavage of ImmR by the metalloprotease ImmA. Thus, transcription is activated and conjugation can take place. **(D)** Upon a distinct concentration (the quorum), the signal peptide PhrI binds to RapI, interfering with its ability to activate ImmA; thus, ImmR can exert repression of the promoter as described in **(A)**.

ICE*Bs*1 is a 20.5-kbp ICE that is found on the chromosome of diverse *B. subtilis* strains. The genes required for conjugative transfer are related to those from ICE*St*1 and Tn*916*. This ICE has one of the highest transfer rates in Firmicutes. ICE*Bs*1 harbors more than 20 ORFs and integrates into a locus coding for a tRNA. ICE*Bs*1 cannot only transfer itself, but it can mobilize non-conjugative plasmids as well (Lee et al., 2012). ICE*Bs*1’s transfer rate was reported to be considerably higher in biofilms, even though the presence of donor cells in a biofilm did not change the frequency of ICE excision (Lécuyer et al., 2018). Interestingly, regulatory processes of ICE*Bs*1 resemble those of plasmid pLS20. In both systems, conjugation is kept in a default “OFF” state by a master regulator that represses expression of the conjugation genes. ImmR is the master regulator of ICE*Bs*1 that can be modulated by the RapI-PhrI cassette, reminiscent of the Rap-Phr module in pLS20 (Figures 5A,B). ImmR inhibits the expression of the excisionase and further downstream genes that are required for ICE*Bs*1 excision and transfer. The RRNPP-family protein RapI induces the production of proteins governing conjugation by interfering with ImmR-mediated repression *via* the anti-repressor ImmA. RapI has been proposed to increase the specific activity of the metalloprotease ImmA that cleaves and thus inactivates ImmR (Bose et al., 2008). In turn, the signaling peptide produced from PhrI inhibits RapI activity. PhrI is encoded downstream of the *rapI* gene within ICE*Bs1* (Auchtung et al., 2005). *phrI* is both expressed from the *rapI* promoter and also produced from its own promoter that is regulated by the sigma factor σ^H^ (McQuade et al., 2001). Thus, *phrI* transcription increases with enhanced cell density. PhrI is secreted and cleaved by host-encoded factors. After pheromone import *via* the oligopeptide permease Opp, PhrI binds RapI, thus inhibiting its activity and subsequently reducing ICE*Bs1* excision and transfer (Auchtung et al., 2005). Similar to iCF10 from pCF10, extracellular concentrations of PhrI correlate with the number of cells harboring ICE*Bs1*. Thus, when only donors harboring ICE*Bs1* are present, PhrI blocks activation of conjugation *via* interaction with RapI. When potential recipient cells without ICE*Bs1* are present, they take up the pheromone PhrI. This then leads to reduced pheromone levels in donor cells, resulting in RapI-dependent activation of excision, *tra*-gene transcription, and consequently conjugative transfer (Auchtung et al., 2005). RapI not only activates conjugative transfer of ICE*Bs*1 but also inhibits sporulation (Auchtung et al., 2005; Bose et al., 2008). De-repression of ICE*Bs*1, followed by excision and conjugative processes, also takes place upon global DNA damage and is mediated by the DNA-repair protein RecA (Auchtung et al., 2005; Bose and Grossman, 2011), the key mediator of the SOS response. It remains elusive, whether RecA can directly influence the activity of the protease ImmA or acts as stabilization factor (Figures 5C,D; Bose and Grossman, 2011).

### pIP501 From *Enterococcus faecalis*

**Figure 6:**
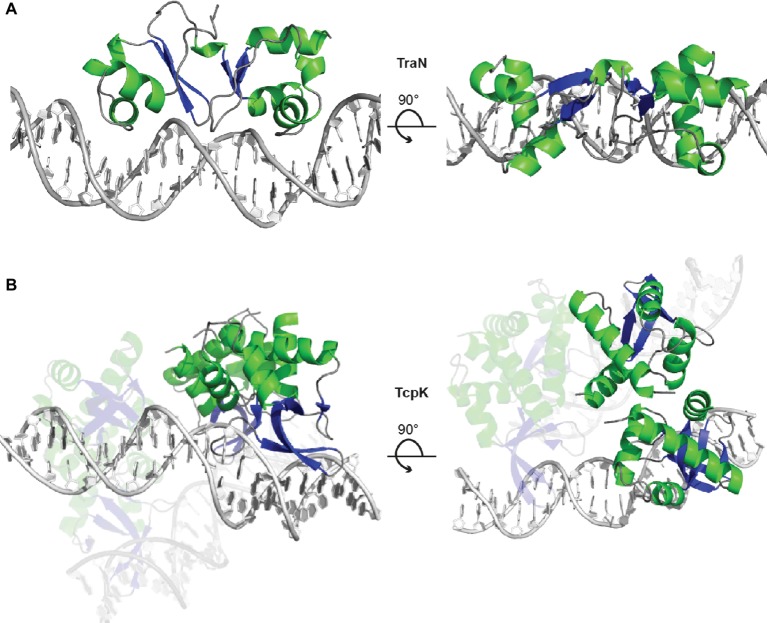
TraN and TcpK are small regulatory proteins exhibiting a winged-helix-turn-helix (wHTH) fold motif. **(A)** TraN is a potent repressor of transcription of the pIP501 *tra*-operon. The TraN protein forms an internal dimer with one wHTH motif in each the N- and C-terminal domain. The TraN-DNA complex (PDB: 6G1T) is shown in cartoon representation, with TraN colored according to secondary structure (α-helices green, β-strands blue) and the DNA in grey. The view is along the recognition helices inserted in the major grooves of the recognition site (left panel) and along the pseudo-two-fold axis of the TraN molecule (right panel). **(B)** The TcpK-DNA complex (PDB: 5VFX) contains two protein dimers bridging two double-stranded DNA molecules with two direct-repeat recognition sequences. The color scheme is the same as in panel **(A),** and the orientation is chosen to show the DNA-binding mode with the β-ribbon of the wing inserted in the major groove of the double-stranded DNA (left panel) and the bridging of the two double-stranded DNA pieces by one of the TcpK dimers (right panel).

**Figure 7:**
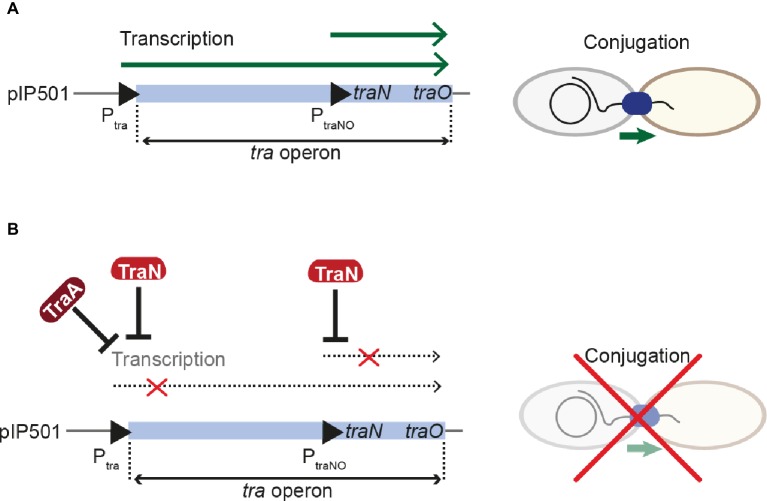
Regulation of pIP501 conjugative transfer. **(A)** Transcription of the pIP501 transfer genes is executed by two promoters, P_tra_ and P_traNO_, leading to the production of factors required for conjugative transfer. **(B)** By a concerted action between the relaxase TraA and TraN, transcription from the P_tra_ promoter is reduced. Further, TraN can repress the P_traNO_ promoter upstream of its own gene. These regulative processes lead to inhibition of conjugative transfer.

The Inc18-family plasmid pIP501 was originally isolated from a clinical *Streptococcus agalactiae* strain and – due to its small size and simplicity – has become the paradigm to study broad host-range plasmids in G+ bacteria with a low G + C content. Inc18 plasmids, including pRE25, pAMß1 and pIP501, have been isolated from clinically relevant *E. faecalis* and *E. faecium* strains and are thought to disseminate resistance to the last-line antimicrobial drug vancomycin, to methicillin-resistant lineages of *Staphylococcus aureus* (Kohler et al., 2018b). The region responsible for conjugative transfer processes is organized as a single operon of 14 kbp, and seven transfer proteins were identified as functional/structural homologs of the G- prototype *A. tumefaciens* T4SS. The *tra*-genes of the pIP501 plasmid were found to be co-transcribed, and mRNA levels remained mostly unchanged until late stationary phase. The relaxase TraA, encoded as the first *tra*-gene of the operon, was described to be leadingly involved in regulation of conjugative transfer. TraA was shown to bind to the P_tra_ promoter, thereby negatively regulating transcription of the *tra*-operon (Kurenbach et al., 2006). Recently, TraN, a small cytosolic transfer protein, was identified as additional repressor of the pIP501 conjugation system by binding to its cognate binding site upstream of the P_tra_ promoter and the *oriT nic-*site (Kohler et al., 2018a). TraN is an internal dimer containing two structurally equivalent domains, which belong to the family of winged-helix fold proteins. Its recognition helices protrude into two adjoining major grooves. The wings are required for formation of the interface between the two domains of the internal dimer and insert into the central minor groove. This composition differs from the DNA binding mode of homo-dimeric winged-helix transcription factors (e.g., LysR-type transcriptional regulators; Figure 6A; Brown et al., 2003). In contrast to the relaxase TraA that shows autoregulation, TraN’s regulatory processes appear to be multi-layered. In addition to the P_tra_ promoter, a second promoter P_traNO_ upstream of the *traN* gene was identified. It was shown to be also negatively regulated by TraN (Figure 7). Thus, it was hypothesized that while the P_tra_ promoter was most likely controlled by a concerted action of the relaxase TraA and TraN, TraN binding to P_traNO_ might not only tune its own production but might also be required to regulate levels of TraO, the proposed surface adhesin needed for contacting potential recipients (Kohler et al., 2018a). Toxicity due to overproduction of surface adhesins was demonstrated for other G+ conjugation systems (Bhatty et al., 2017). Nevertheless, the nature of the signal either from potential recipients and/or the environment preceding the nicking of the plasmid DNA by TraA has not been identified so far. Since TraN-homologs and potential TraN binding sites were identified on several Inc18-like and other related multi-resistance plasmids, a similar mechanism of repression was postulated for those plasmids highlighting the potential applicability of TraN as a pharmacological target to combat the dissemination of antibiotic resistances (Kohler et al., 2018a).

### pCW3 From *Clostridium perfringens*

The tetracycline resistance plasmid pCW3 from *Clostridium perfringens* belongs to a class of related antibiotic resistance and toxin plasmids (Li et al., 2013). The transfer of clostridial plasmids (*tcp*) locus encoding 11 genes mediates conjugative transfer, with eight proteins essential for these processes (Wisniewski and Rood, 2017). Recently, the structure of TcpK was solved, an essential protein encoded upstream of the *oriT* and shown to be involved in efficient conjugation of pCW3 (Traore et al., 2018). Similar to TraN from pIP501, TcpK is a member of the winged helix-turn-helix protein family and binds specifically to tandem repeats within the pCW3 *oriT*. The complex structure of TcpK with its binding site DNA revealed a binding mode, completely different from other known winged helix-turn-helix proteins. Interestingly, while the wing protrudes into the major groove, the recognition helix makes only a single contact to the binding site DNA. Further, each TcpK dimer binds two binding boxes on different DNA molecules, thus suggesting that TcpK dimers bridge across two DNA molecules (Figure 6B). It was suggested that TcpK is an accessory factor of the pCW3 relaxosome, most likely binding to the *oriT* by directly interacting with sequences only present in this region of the plasmid, thus aiding in the proper recruitment of the relaxase TcpM (Traore et al., 2018). Even though the binding mode of TcpK has been uncovered and described in extensive detail, the exact mechanism of regulation remains to be elucidated.

### pMV158 From *Streptococcus agalactiae*

pMV158 is a rolling-circle plasmid and was originally isolated from *S. agalactiae* (Burdett, 1980). This 5.5-kbp plasmid is not conjugative but can be mobilized among diverse G+ and G- species by several Inc18-family plasmids including pIP501 and pAMß1 (Priebe and Lacks, 1989; Van der Lelie et al., 1990; Grohmann et al., 1999). MobM is the relaxase of pMV158 and belongs to the MOB_V_ family of relaxases (Garcillán-Barcia et al., 2009). The full-length MobM as well as the relaxase domain specifically bind to *oriT*_pMV158_ and are able to perform ss-cleavage of supercoiled pMV158 DNA at the *nic*-site (Grohmann et al., 1999; de Antonio et al., 2004; Lorenzo-Díaz et al., 2011, 2014). Recently, the structure of MobM in complex with different DNA substrates was solved (Pluta et al., 2017). The structures reveal a tight network of protein-DNA interactions involving base-specific as well as backbone interactions. Besides its role in mobilization, MobM is able to repress its own transcription by binding to the *oriT* region, which contains two promoter sequences, one directly overlapping the *oriT* and the second adjacent to the *oriT* (Lorenzo-Díaz et al., 2012), in a similar mode as observed for TraA of pIP501 (Kurenbach et al., 2006). MobM not only autoregulates its own synthesis but is also involved in regulating the pMV158 copy number by binding to the promoter region of the antisense RNAII consequently alleviating the repression of the replication initiator RepB (Lorenzo-Díaz et al., 2017).

### pSK41 From *Staphylococcus aureus*

**Figure 8:**
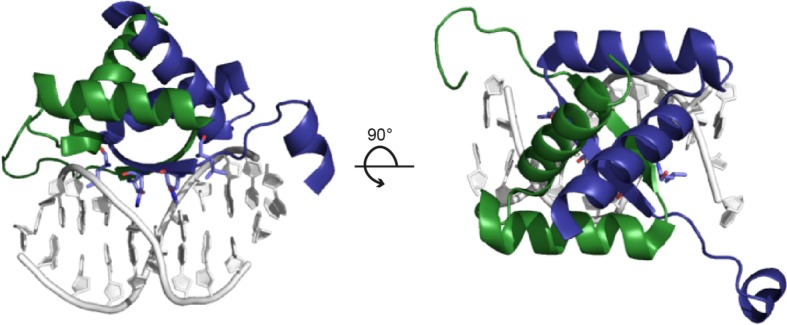
Structure of the ArtA-DNA complex (PDB: 3GXQ). ArtA, the master regulator of the plasmid pSK41, exhibits a ribbon-helix-helix fold. The dimeric repressor is shown in cartoon representation, with chains A and B colored in green and blue, respectively. The anti-parallel β-ribbon permeates the major groove of the binding-site DNA and exerts base-specific interactions (left panel). The view along the twofold axis of the ArtA dimer and the pseudo-palindromic double-stranded DNA site is shown in the right panel.

pSK41 and pGO1 are two well-characterized representatives of a large family of low-copy number multi-resistance plasmids from *S. aureus* (Liu et al., 2013). pSK41-like plasmids are self-transmissible and comprise a compact conjugation system with a 14-kbp *tra* region, which consists of two operons, *traA-K* and *traL-M* (Firth et al., 1993), and an additional, divergently transcribed gene, *artA (trsN* in pGO1), which will be discussed in detail below (Ni et al., 2009). However, in contrast to the architecture of Inc18-like plasmids, encoding the relaxase as the first open reading frame of the operon, the relaxase gene of pSK41-like plasmids is outside of the *tra* region. The product of the conversely oriented gene, ArtA, is a global transcriptional regulator of pSK41 with six binding sites present on the plasmid, repressing the transcription of conjugative genes as well as those of the segregation system. All three *tra* promoters (P*_artA_*, P*_traA_*, and P*_traL_*) contain these specific ArtA recognition sites and exhibit ArtA binding affinities in the nanomolar range. The crystal structure of ArtA in complex with its cognate DNA binding site reveals that ArtA belongs to the family of ribbon-helix-helix DNA-binding proteins with a lysine-rich N-terminal stretch, which supposedly contributes to additional binding strength (Figure 8; Ni et al., 2009).

### Other Mobile Genetic Elements

ICEs similar to ICE*Bs*1 include Tn*916*, ICE*St*1, and ICE*St*3. Tn*916* shows a wide host range and is frequently encountered in clinical isolates of *E. faecalis*, *Clostridium difficile*, and *Streptococcus pneumoniae* (Roberts and Mullany, 2009). This ICE is one of the smallest and least complex conjugative elements and carries a tetracycline resistance gene. It can replicate autonomously in *B. subtilis*, which depends on its relaxase. Tn*916* shows its maximal excision frequency during late exponential phase (Celli et al., 1997), thus activation of the element depends on the respective growth phase. Circularization of Tn*916* is required for conjugative transfer, since distinct transcripts can only be produced when the *att* sites are covalently joined (Celli and Trieu-Cuot, 1998). Interestingly, when exposed to tetracycline conjugative transfer frequencies increase 19-fold in *B. subtilis*, while excision frequencies were apparently not affected (Showsh and Andrews, 1992; Celli et al., 1997).

ICE*St*1 with a size of 35 kbp and ICE*St*3 with 28 kbp are closely related ICEs that are found in streptococci. While ICE*St*3 was shown to be transferred to other species including *S. pyogenes* and *E. faecalis*, it is still a matter of debate whether ICE*St*1 is functional or was acquired by transformation (Bellanger et al., 2009; Fontaine et al., 2010). The dissemination module of these ICEs is a 14-kbp polycistronic operon under the control of the P_cr_ promoter (Carraro et al., 2011). It is suggested that the mobility of this ICE family relies on the activity of the P_cr_ promoter (Carraro et al., 2011). The actual DNA-processing machinery is suggested to involve a putative relaxase, which, in addition to the *oriT*, seems to be conserved between these ICEs, ICE*Bs*1 and Tn*916* (Jaworski and Clewell, 1995; Burrus et al., 2002; Rocco and Churchward, 2006). The *arp2* gene encodes a protein reminiscent of the master-regulator ImmR of ICE*Bs*1, while *orfQ* might encode an ImmA-like metalloprotease. Interestingly, while ICEs are usually controlled by one central repressor belonging to unrelated families, cI or ImmR, ICE*St*1/ICE*St*3 family members harbor both repressors (Bellanger et al., 2009; Carraro et al., 2011).

## Conclusions and Perspectives

Assembly and operation of multiprotein complexes such as bacterial conjugation systems require large amounts of energy, provided by one or more ATPases encoded by the conjugation system itself. To minimize energy costs of DNA-protein complex transport, expression and/or activity of single crucial components or the whole conjugation system need to be tightly controlled. This is exerted at different levels by different modes (1) at the transcriptional level by controlling the expression of components, such as the conjugative relaxase and/or accessory relaxosome components, or by controlling the production of surface adhesins required for contacting potential recipient cells, (2) *via* cell density sensing (QS) and sex pheromone-induced surface adhesin production, or (3) *via* a master regulator encoded by the plasmid or ICE itself that keeps the conjugative process in a default “OFF” state until the respective antirepressor gets activated, which turns “ON” the conjugative process.

Some plasmids or ICEs dispose only one of these regulatory modes, and others use combinations of them to maintain conjugative activity at an optimum level. Additionally, conjugative processes of some plasmids as well as ICEs are highly growth-phase dependent, with transfer taking place exclusively during exponential growth or exhibiting maximum rates only during (late) exponential growth. Several well-known conjugative plasmids and ICEs of G+ origin use pheromone-sensor receptors of the RRNPP family to regulate the conjugation process. Although all members of the RRNPP family display remarkable structural similarity, the downstream reactions often differ widely. Therefore, even though construction of pheromone mimicries would be doable based on the extensive information gathered for this protein family, their structural similarity combined with their differing effects on conjugation would cause problems. Precise data on the regulatory mechanism combined with extensive testing of potential pheromone analogues for diverse conjugation systems would be a first step to solve this problem.

Conjugation is one of the most important means in the dissemination of antibiotic resistance and virulence factors among pathogenic bacteria. Thus, elucidating mechanistic and regulatory details of these large nanomachines is crucial for developing novel approaches to combat multi-resistant pathogens: Important advancements in this direction have been made recently by solution of the cryo-EM structure of the first bacteria-killing T4SS core complex from the G- phytopathogen *Xanthomonas citri* (Sgro et al., 2018) and by the experimental proof that the relaxase has to unfold for efficient translocation through the conjugative T4SS complex (Trokter and Waksman, 2018). Although the abundance of ICEs seems to largely exceed that of conjugative plasmids (Guédon et al., 2017), mechanistic details of their transfer remain elusive. Thus, the aim of future research should lie on the elucidation of the spreading mechanism of ICEs to enable the development/design of specific inhibitors reducing their dissemination.

## Author Contributions

VK, WK, and EG drafted the manuscript. VK designed the figures. All authors approved the final version of the manuscript.

### Conflict of Interest Statement

The authors declare that the research was conducted in the absence of any commercial or financial relationships that could be construed as a potential conflict of interest.
